# The anthropogenic imprint on temperate and boreal forest demography and carbon turnover

**DOI:** 10.1111/geb.13773

**Published:** 2023-10-16

**Authors:** Thomas A. M. Pugh, Rupert Seidl, Daijun Liu, Mats Lindeskog, Louise P. Chini, Cornelius Senf

**Affiliations:** ^1^ Department of Physical Geography and Ecosystem Science Lund University Lund Sweden; ^2^ School of Geography, Earth and Environmental Science University of Birmingham Birmingham UK; ^3^ Birmingham Institute of Forest Research University of Birmingham Birmingham UK; ^4^ Ecosystem dynamics and forest management group Technical University of Munich Freising Germany; ^5^ Berchtesgaden National Park Berchtesgaden Germany; ^6^ Department of Botany and Biodiversity Research University of Vienna Vienna Austria; ^7^ Department of Geographical Sciences University of Maryland College Park Maryland USA

**Keywords:** carbon cycle, forest demography, forest disturbance, forest dynamics, harvest, land use

## Abstract

**Aim:**

The sweeping transformation of the biosphere by humans over the last millennia leaves only limited windows into its natural state. Much of the forests that dominated temperate and southern boreal regions have been lost and those that remain typically bear a strong imprint of forestry activities and past land‐use change, which have changed forest age structure and composition. Here, we ask how would the dynamics, structure and function of temperate and boreal forests differ in the absence of forestry and the legacies of land‐use change?

**Location:**

Global.

**Time Period:**

2001–2014, integrating over the legacy of disturbance events from 1875 to 2014.

**Major Taxa Studied:**

Trees.

**Methods:**

We constructed an empirical model of natural disturbance probability as a function of community traits and climate, based on observed disturbance rate and form across 77 protected forest landscapes distributed across three continents. Coupling this within a dynamic vegetation model simulating forest composition and structure, we generated estimates of stand‐replacing disturbance return intervals in the absence of forestry for northern hemisphere temperate and boreal forests. We then applied this model to calculate forest stand age structure and carbon turnover rates.

**Results:**

Comparison with observed disturbance rates revealed human activities to have almost halved the median return interval of stand‐replacing disturbances across temperate forest, with more moderate changes in the boreal region. The resulting forests are typically much younger, especially in northern Europe and south‐eastern North America, resulting in a 32% reduction in vegetation carbon turnover time across temperate forests and a 7% reduction for boreal forests.

**Conclusions:**

The current northern hemisphere temperate forest age structure is dramatically out of equilibrium with its natural disturbance regimes. Shifts towards more nature‐based approaches to forest policy and management should more explicitly consider the current disturbance surplus, as it substantially impacts carbon dynamics and litter (including deadwood) stocks.

## 1 INTRODUCTION

The vast majority of temperate forests and much of the boreal forest have been heavily transformed by human activities. Of the remaining forested areas, only 10% of northern hemisphere temperate and southern boreal forests and 64% of northern boreal forests are considered to be intact (Potapov et al., 2017). Much of the human impact is driven by harvest, which is the dominant form of disturbance across temperate and southern boreal forests (Curtis et al., 2018). Demand for wood products, alongside efforts to increase productivity, has also transformed the species composition of large areas of forest. These activities have resulted in forests which are relatively young (McDowell et al., 2020), often composed of non‐native species mixtures, and contain an unnaturally high level of monocultures in many regions (Forest Europe, 2020). Such changes in age structure have been compounded by legacies of past land use, resulting in some regions in a disproportionate amount of relatively young forest, regrowing on former agricultural land (Hurtt et al., 2020; Winkler et al., 2021). These changes in forest composition and age structure in turn affect the form, severity and frequency of natural disturbances to which these forests are subjected (Rich et al., 2007; Seidl et al., 2016, 2017). Natural disturbances, by agents such as windthrow, wildfire, insects or pathogens, further feedback on forest composition and age structure and influence how those forests are managed (e.g. salvage and sanitation logging).

The widespread human transformation of temperate and boreal forests alters the services provided by forests. It is the large trees that are found in older forest stands that disproportionately store more carbon (Enquist et al., 2020), provide keystone habitats for other species (Lindenmayer et al., 2014) and typically have high cultural and amenity value (Blicharska & Mikusiński, 2014). Conversely, younger forest stands are often highly productive and current imbalances between forest age structure and disturbance rate are acting to substantially increase the terrestrial carbon sink (Pugh, Lindeskog, et al., 2019). Similarly, relatively young, productive plantations are the mainstay of the global wood products industry. Both young and old forest stands can have high deadwood stocks, supporting biodiversity, but the rate of disturbance and whether dead wood is harvested will influence these stocks. In the context of a major discourse relating to a targeted increase in the global forest area (Doelman et al., 2020; Trillion Trees, 2021; United Nations, 2017) and the restoration and conservation of ‘natural’ conditions (Büscher et al., 2017; Wild et al., 2020; Wilson, 2016), understanding the form and function of forests that would naturally grow in any given location can provide important input to decisions around where and if such policies may be particularly valuable. Furthermore, the ability to quantify the dynamics of natural forests is key to calculations of historical changes in the Earth system, including accurate estimates of land‐use change emissions (Li et al., 2017) and carbon storage potentials (Erb et al., 2018). These historical changes provide the baseline for Earth system model simulations exploring future climate (Lawrence et al., 2016).

Our capability to observe the current, transformed, state of our forests has improved dramatically in recent years, in terms of both size and age structure (Los et al., 2012; Potapov et al., 2021; Sexton et al., 2013; Simard et al., 2011) and rates of change therein (Hansen et al., 2013; Senf & Seidl, 2021a). However, we have precious little information on the large‐scale age structure and function of temperate and boreal forests in the absence of forestry and the legacies of past land use; the imprint of these anthropogenic activities at the biome scale is largely unknown. In principle, the latest large‐scale demographic vegetation models, which explicitly represent forest structure, should be well‐placed to provide such quantifications (Fisher et al., 2018; Pugh, Lindeskog, et al., 2019). However, assessing the age structure and carbon cycling without the current anthropogenic imprint requires inferring the natural disturbance regime (Bengtsson et al., 2000; Lorimer, 1989; Pflugmacher et al., 2012) and these large‐scale models currently lack appropriate modules to simulate natural disturbances (Pugh et al., 2020). Here, we develop a lightweight empirical model of forest disturbances for northern temperate and boreal forests and couple it within a demographic vegetation model. We then use this new tool to answer the questions: (1) To what extent do forests currently suffer from a surplus or a deficit of disturbance (from both human and natural causes) relative to their state in the absence of forestry and past land‐use legacies? (2) How has this changed forest age structure and what are the implications for forest carbon stocks and turnover?

## 2 METHODS

**FIGURE 1:**
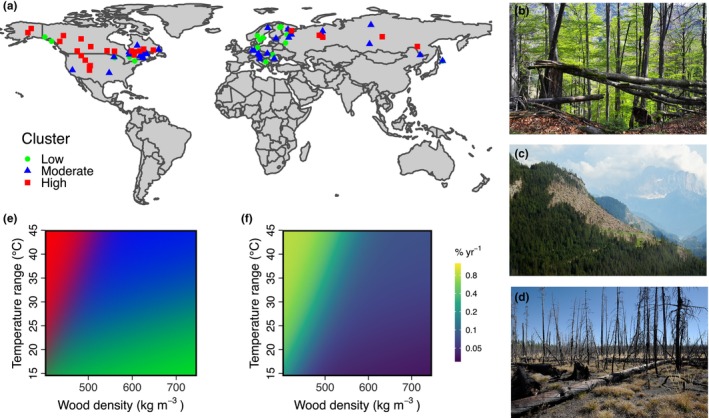
(a) Location of the 77 temperate and boreal forest landscapes used as reference for natural disturbance dynamics in this study, with colour and shape indicating the disturbance activity cluster type (low, moderate and high disturbance activity). (b–d) Examples of landscapes within each disturbance activity cluster, with a landscape dominated by low‐severity windthrow and gap‐type dynamics (b; Photo by R. Seidl), a landscape affected by moderate‐severity windthrow (c; Photo by C. Senf) and a landscape affected by high‐severity fire (d; Photo by R. Seidl). (e) Likelihood of a landscape originating from one of the three disturbance activity clusters as a function of community mean wood density and of 30‐year mean annual temperature range. (f) Characteristic mean disturbance rates as a function of community mean wood density and of 30‐year mean annual temperature range. The disturbance rates are estimated based on the likelihood that a forest, in its current state, is subject to low, moderate or high disturbance activity (see e) and based on the average disturbance rates of each disturbance activity cluster (see Table 2). Disturbance rates thus form a continuum between asymptotic rates for the low and high activity clusters. Note that disturbance rates in (f) are on log_10_‐scale.

**FIGURE 2:**
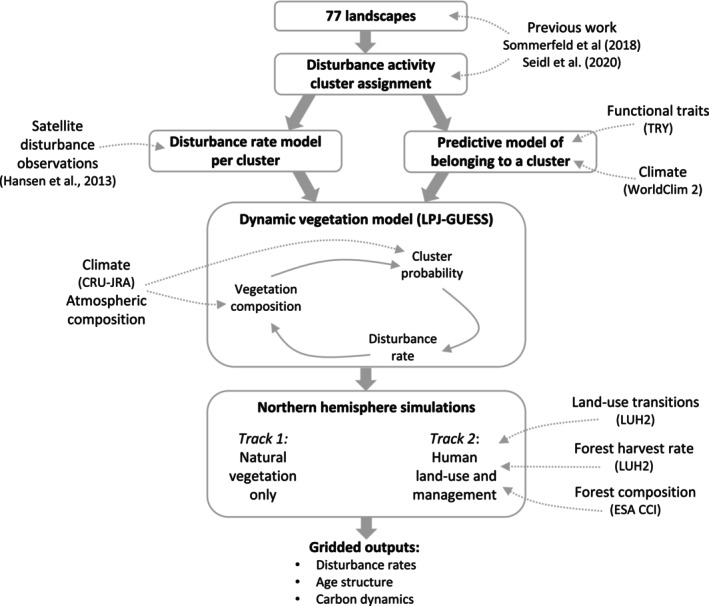
Flow chart of the methodology used in this study. External data sources are denoted with dotted arrows (see text for detail of sources). The cluster probability model is implemented directly inside LPJ‐GUESS, based on the model simulated vegetation composition, and the resultant disturbance rate feeds back on the simulated vegetation composition.

We used a novel fusion of satellite observations of stand‐replacing disturbances in 77 protected areas (i.e. landscapes of forest development without human intervention; Figure 1a), statistical analysis and dynamic vegetation modelling, to generate wall‐to‐wall estimates of natural disturbance frequency across northern hemisphere temperate and boreal forests. We first developed an empirical model of natural disturbance rates from agents including fire, windthrow and insects/pathogens, but excluding management, for temperate and boreal forests. We then assessed how average disturbance rates were related to community mean functional traits and climate variability, linking empirical disturbance relationships to the vegetation projected by a dynamic global vegetation model. We based this on a recent finding that natural disturbances of temperate and boreal forests can be consistently categorized into three distinct clusters of disturbance activity (Seidl et al., 2020; Sommerfeld et al., 2018), parsimoniously describing the prevailing disturbance regime (Figure 1a). We used the LPJ‐GUESS dynamic vegetation model (Smith et al., 2014), which explicitly simulates plant functional types covering different successional stages, to simulate forest functional composition in the absence of human management. We interactively coupled this simulation to the empirical disturbance models described above to generate estimates of natural disturbance rates across all northern hemisphere temperate and boreal forests. Comparing this result to a recent estimate of actual disturbance rates over 2000–2014 (including both natural and anthropogenic disturbances) allowed to identify which areas of forest are in disturbance surplus or deficit in relation to their natural disturbance regime. Finally, we combine our natural disturbance rate estimates with reconstructions of human impact on forests (Hurtt et al., 2020) to test how anthropogenic changes to disturbance rate have altered the age structure of temperate and boreal forests and their carbon turnover time. The methodological flow is summarized in Figure 2.

### 2.1 Empirical model of disturbance rates

We built an empirical model of disturbance rates based on a set of 103 strictly protected landscapes (Seidl et al., 2020) and globally available forest canopy disturbance maps for the period 2001 to 2014 (Hansen et al., 2013). The forest disturbance maps of Hansen et al. were derived from satellite time series of the Landsat archive and depict stand‐replacing changes in the top tree canopy at a spatial grain of 30 m. As southern hemisphere forests follow very different functional strategies to those in the northern hemisphere, resulting in different mean disturbance rates, we excluded all landscapes in the global south (total of 22 landscapes). We further excluded four landscapes that were primarily dominated by ephemeral defoliation, all of which were located in northern Norway (Seidl et al., 2020). Our final sample thus consisted of 77 landscapes (Figure 1a), from which we built our empirical disturbance rates model. Whilst the time series length of 14 years is very short to characterize disturbance regimes, we here rely on a space‐for‐time substitution effect. That is, as all our landscapes are independent, the total length of observation sums to 1078 years. Across all landscapes, there was a total forest area of 15,440,181 ha, and all landscapes were unmanaged during the reference period (e.g. core zones of national parks).

For each landscape, we extracted disturbance and forest cover maps from Hansen et al. (2013) and calculated the annual number of pixels flagged as disturbed and the total number of pixels flagged as forest (using a cut‐off of >10% forest cover per pixel for both). We used a binomial model with logit link function and random variation in the intercept among landscapes and years to estimate the average annual disturbance rate. We further split the calculation of disturbance rates into three distinct clusters of disturbance activity (termed low, moderate and high disturbance activity) following previous research on global disturbance patterns (Seidl et al., 2020; Sommerfeld et al., 2018). Membership in a disturbance activity cluster was based on the shape and configuration of the disturbance patches and is primarily related to the prevailing disturbance agent within a landscape: The low disturbance activity cluster is dominated by small‐scale windthrow and pathogens, whereas the moderate disturbance activity cluster is dominated by large‐scale windthrow and bark beetle outbreaks, and the high disturbance activity cluster is dominated by wildfire (Seidl et al., 2020; Sommerfeld et al., 2018). From the model, we derived random draws, including parameter uncertainty (fixed and random) and model uncertainty, from which we finally calculated average disturbance rates for each disturbance activity cluster. The calculation of disturbance rates is hence not based on the raw data, but on random draws from the model (of which the observed data might be one realization). Our approach consequently allows for some surprise of large disturbances not observed in the raw data.

We also calculated disturbance rates based on a definition of closed‐canopy forest as used in Pugh, Arneth, et al. (2019), instead of using a 10% canopy cover cut‐off per Landsat pixel, in order to facilitate a comparison with results from their analysis. In this definition, forest area was designated as all 0.01° grid cells with at least 50% canopy cover, whilst disturbed forest was the sum of all loss pixels within those forest area grid cells. This more conservative definition of forest results in a much smaller overall forest area.

To provide a link between the average disturbance rates per disturbance activity cluster and global dynamic vegetation models, we built empirical models predicting the probability of a simulated forest to belong to a certain disturbance activity cluster based on temperature and precipitation averages and ranges, community‐weighted mean wood density, community‐weighted maximum tree height and the share of conifer species (Table S1). Predictors of cluster membership were based on previous studies showing their importance for discriminating between different disturbance regimes (Sommerfeld et al., 2018). We used a parametric multinomial classification model (Venables & Ripley, 2002) for modelling the disturbance activity cluster membership, instead of a more complex machine learning model, as a parametric model allows for easy implementation in dynamic global vegetation models. We trained the model based on the 77 landscapes, for which climatic data for average annual temperature and total annual precipitation were extracted from the global WorldClim 2 bioclimatic variables database covering the climate normal period 1980 to 2010 (Fick & Hijmans, 2017). Trait values were derived from the TRY database (Kattge et al., 2020), with genus averages used to gap‐fill values for the 23 species (17%) for which trait information was not available. Community‐weighted traits were calculated by the share of each species in terms of landscape coverage, which was estimated from local experts for each landscape (Sommerfeld et al., 2018). We finally identified the most parsimonious subsets of predictor values using the Akaike Information Criterion; that is, we identified the model that yielded best predictive performance with fewest predictors. We then predicted the probability of belonging to any of the three clusters using the final model, from which we could derive a weighted average disturbance rate (using the probabilities as weights), providing a simple and continuous empirical link of stand type and climate to disturbance probability.

We did not consider any sensitivities of disturbances to climate variability in our model, despite recent findings of a consistent climate response of natural disturbances across temperate and boreal forests (Seidl et al., 2020; Senf & Seidl, 2018). We decided not to do so because we were mainly interested in the long‐term average disturbance rates, instead of the annual variability; and because it is challenging to extrapolate locally fitted regression‐based response curves outside their known data space.

### 2.2 Vegetation model simulations

Simulations were based on the LPJ‐GUESS dynamic global vegetation model v4.0 subversion revision 8139 (Smith et al., 2014). LPJ‐GUESS was designed to explicitly simulate the demography of forests, including both spatial variation across the landscape due to stand‐replacing disturbance events and vertical structure and competition of trees of different ages and plant functional types (PFT; Smith et al., 2001, 2014). LPJ‐GUESS simulates spatial variation in forests in each grid cell through a series of replica 1000 m^2^ patches (here 20 as standard). All patches in a grid cell are forced by the same environmental boundary conditions but are subject to stochastic disturbances with a characteristic return interval, which kill all trees in the patch. In previous studies, this return interval has typically been defined as a fixed value across all grid cells of 100 or 200 years (Hickler et al., 2012; Smith et al., 2014), although more recently, satellite observations have been used to specify grid‐cell‐specific intervals (Pugh, Arneth, et al., 2019). Trees within a patch are simulated based on age cohorts. This combination of horizontal structure of disturbance patches across the landscape and vertical structure within the canopy of a single stand allows LPJ‐GUESS to simulate tree size distributions characteristic of real forests (Smith et al., 2014).

LPJ‐GUESS was modified to replace the default fixed disturbance return interval with a dynamic calculation of return interval using the empirical model described in Section 2.1, allowing direct feedback between forest composition and disturbance rates (Figure 2). The probability of a particular patch belonging to a low, moderate or high disturbance activity cluster was based upon annual temperature range and simulated patch‐mean wood density across all PFTs and age cohorts in the patch. Wood density was set at the PFT level, and patch means were weighted according to the woody biomass of each individual. For maximum height, means were weighted according to the crown‐projection area of each individual. The annual temperature range was taken from the driving climate data as a mean over the 30 preceding years. Disturbance was not permitted to occur twice within a 10‐year period. Disturbance return intervals in LPJ‐GUESS were capped at 1000 years as uncertainty increases as events become very rare and because beyond 1000 years the influence on forest biomass is minimal (Pugh, Arneth, et al., 2019). All carbon from vegetation which was naturally disturbed was transferred to the litter, from whence it could be respired to the atmosphere or broken down into soil.

Wood density and maximum height values at the PFT level were assigned based on the composition of the protected landscapes. Tree species occurring in any of the landscapes were assigned to an LPJ‐GUESS PFT based on leaf type, deciduousness and shade‐tolerance (Table S5). Mean values of each trait for each PFT were then calculated based on the relative abundance of each species across the different landscapes (Figure S2), using information compiled by Sommerfeld et al. (2018) and Seidl et al. (2020). These trait values were only used for the calculation of disturbance rate. In addition to the new disturbance module, we also updated the maximum age parameters for boreal PFTs based on the literature. The shade‐intolerant broadleaf PFT was assigned a maximum age of 150 years, representing a compromise between the ca. 100 years reported for North America and ca. 200 years reported for Siberia (Kneeshaw & Gauthier, 2003; Shorohova et al., 2011). The maximum age for boreal evergreen needleleaf PFTs was set to 300 years based on Shorohova et al. (2009). The modified LPJ‐GUESS is archived as Subversion revision 12,260.

Two sets of simulations were made using the new dynamic disturbance module (Table 1), those simulating natural vegetation everywhere and those including human land‐use change and forest harvest. For the natural vegetation everywhere case, LPJ‐GUESS was allowed to select the mix of vegetation that was most successful at that location through its usual process of competition (NatPmid). Two sensitivity simulations explored the effect of using disturbance rates 2 standard errors below (NatPlow) or above (NatPhigh) the mean disturbance rate for each cluster. In addition, one further sensitivity simulation explored the effect of calculating disturbance rates using the closed‐canopy forest definition (NatCmid).

**TABLE 1:** LPJ‐GUESS simulations made as part of this study.

Code	Disturbance rate	Forest definition used for disturbance rate		Human land use
		Pixel	Closed‐canopy	
NatPmid	Mean	x		
NatPlow	Mean – 2 S.E.	x		
NatPhigh	Mean + 2 S.E.	x		
NatCmid	Mean		x	
LUHPmid	Mean	x		Standard
LUHPlow	Mean	x		Lowest
LUHPhigh	Mean	x		Highest

To include past human land‐use legacies and forest harvest from past to present, we forced LPJ‐GUESS with the LUH2 land‐use change and management dataset (Hurtt et al., 2020). We used the gross land‐use transitions dataset from LUH2, which specifies every land‐use transition between forest, cropland and pastureland, as well as rates of forest harvest, as previously applied in Pugh, Lindeskog, et al. (2019). In order to account for legacy effects of land‐use change and harvest prior to 1901 on soil and litter carbon stocks, land‐use change and harvest was applied during the spin‐up period from the year 1700 onwards. LUH2 specifies land use, including whether a forest is primary (i.e. not harvested, cut or converted since the year 850 CE) or secondary, but does not provide information on forest species composition.

In order to capture the change in forest composition that has resulted from human management, we constrained the type of tree species that was allowed to establish within forested lands in the LUH2‐forced simulations (LUH2 primary and secondary forest categories) according to the dominant forest type recorded in ESA CCI land cover for the year 2015 (ESA, 2017). For LPJ‐GUESS grid cells defined as broadleaf dominated in ESA CCI (>80% of all forest pixels classified as broadleaf), only broadleaf PFTs were allowed to established and vice versa for grid cells defined as needleleaf (>80% classified as needleleaf). In mixed pixels (all other forested areas), all PFTs were allowed to establish. In land‐use change events, rules were adopted to govern which ages of forest should be preferentially subjected to transitions. When converting forest to cropland or pasture, the oldest forest stands were preferentially cut when converting the LUH2 category ‘primary’ forest. When converting the LUH2 category ‘secondary’ forest, young forest stands were preferentially cut. In wood harvest events, we preferentially cut old stands (LUH2 category ‘primary’), then old secondary stands (‘secondary old’) and finally young stands (‘secondary young’).

Natural disturbances were allowed to affect all forests in the LUH2‐forced simulations, just as in the natural vegetation simulations. In addition to the standard LUH2‐forced simulation (LUHPmid), we ran two extra sensitivity simulations to assess uncertainty using low (LUHPlow) and high (LUHPhigh) land‐use change estimates from the same database (Hurtt et al., 2020).

All LPJ‐GUESS simulations were forced by CRU‐NCEP climate and observed atmospheric CO_2_ mixing ratios for the period 1901–2015 (Le Quéré et al., 2016). Atmospheric nitrogen deposition rate was taken from Lamarque et al. (2013). Prior to 1901, the model was spun up from bare ground for a period of 1500 years in order to allow vegetation and soil pools to come into equilibrium. The spatial resolution of the simulations was 0.5° × 0.5°. LPJ‐GUESS contains a process‐based fire model, which was turned off to avoid double counting with the simple disturbance model used here.

Calculations for carbon cycle variables are presented as averages over 2001–2014. Carbon turnover time was calculated as carbon mass (*CM*) divided by net primary productivity (*NPP*) summed across all *n* grid cells in a biome, as follows,

(1) $$\tau =\frac{\sum_{i=1}^{i=n}{\mathrm{CM}}_{i}\;A_{i}}{\sum_{i=1}^{i=n}{\mathrm{NPP}}_{i}\;A_{i}},$$

where *A* is the area of forest and *i* is a reference for a specific grid cell. Ecosystem carbon turnover time was based on total carbon mass across vegetation, litter (which includes all recently dead plant material, whether deadwood, leaves or roots) and soil, whilst vegetation carbon turnover time only used carbon mass in vegetation. In addition, we also calculated grid‐cell‐level statistics in which τ=CM/NPP was calculated for each grid cell and forest‐area‐weighted statistics were then calculated at the biome level. LPJ‐GUESS does not directly simulate biomes, but rather simulates PFTs; therefore, to enable to comparison of biome distribution in LPJ‐GUESS with observation‐based sources (Haxeltine & Prentice, 1996; Hengl et al., 2018), a set of rules based on the leaf area index of the different PFTs were used (Table S3), based on those in Smith et al. (Smith et al., 2014).

In addition to outputting disturbance rate and carbon variables, LPJ‐GUESS also tracked changes in forest age structure resulting from both natural and anthropogenic disturbances and from land‐use change. Age structure presented is that simulated for the year 2014. The age structure was output in 10‐year age bins up to 140 years, using the same 140 year cut‐off point between young and old forest stands as employed in Pugh, Lindeskog, et al. (2019).

### 2.3 Masking

All results were masked such that each grid cell lay within the temperate or boreal forest biomes in the northern hemisphere, as defined by the biomes dataset of Olson et al. (2001). For maps, only grid cells with a simulated biomass density in the LPJ‐GUESS baseline simulation (NatPmid, Table 1) of at least 1 kg C m^−2^ and a minimum of 10% canopy cover based on Hansen et al. (2013) are shown. For closed‐canopy forest calculations, this threshold was set to 5% closed‐canopy forest cover, following Pugh, Arneth, et al. (2019). Calculations for forest age and carbon cycle are based on the full forest area, as defined by the LUH2 primary and secondary forest cover fractions averaged over 2001–2014.

### 2.4 Evaluation

We carried out an evaluation of the ability of LPJ‐GUESS to simulate vegetation biomass in the protected landscapes. We used aboveground biomass data from ESA Biomass CCI v3.0 for the year 2010 (Santoro & Cartus, 2021). We selected all 100 m resolution pixels which lay within the boundaries of each landscape and calculated the mean biomass value across the landscape, excluding any pixels with zero biomass values, which were assumed to be non‐forest areas such as lakes. We converted biomass to carbon using a conversion factor of 0.5 kg C kg^−1^ DM and converted aboveground biomass to total biomass (as simulated by LPJ‐GUESS) by dividing by a factor of 0.78 (Ma et al., 2021). We extracted the LPJ‐GUESS grid cells that covered the landscapes, averaging biomass values over multiple grid cells in the event that the landscape was covered by more than one.

We also carried out an assessment of the ability of LPJ‐GUESS to represent succession in the boreal region, where our simulations are most sensitive to the interaction between succession and disturbance frequency. We compiled information on the typical time of transition of species dominance from field observations in nine different regions located across North America and Eurasia (Bergeron & Fenton, 2012; Heinselman, 1981; Lecomte & Bergeron, 2005; Shorohova et al., 2009; Taylor et al., 2020; van Cleve & Viereck, 1981). We then ran LPJ‐GUESS simulations at these locations with a length of 300 years, with all patches subject to a stand‐replacing disturbance at the end of the spin‐up period and the vegetation allowed to recover from bare ground, without further stand‐replacing disturbances. All environmental forcings were kept as during the spin‐up period throughout in order to not confound succession processes with environmental trends. Details of the locations are provided in the Supplementary Information, Section S1.

## 3 RESULTS AND DISCUSSION

### 3.1 Modelling disturbance rates and clusters across 77 landscapes

Our results provide a consistent empirical model of natural disturbance rates across the temperate and boreal forests of the Northern Hemisphere. The rate of stand‐replacing disturbances in the 77 protected areas, defined as near total loss of canopy cover for an area of ca. 0.1 ha or larger (Frolking et al., 2009; Hansen et al., 2013), varied substantially between landscapes (Figure 1). For landscapes of low disturbance activity, resulting mainly from small‐scale windthrow and pathogens, the average rate was 0.03 ± 0.07% of forest area disturbed per year (average and standard deviation calculated from 10,000 random draws from the model; Table 2). For landscapes of moderate disturbance activity, resulting mainly from windthrow and bark beetle outbreaks, the average disturbance rate was 0.08 ± 0.19% yr^−1^ (Table 2). For landscapes of high disturbance activity, which was mostly related to fire, the average disturbance rate was 0.86 ± 1.80% yr^‐−1^ (Table 2). The disturbance activity clusters alone explained 25% of the variability in observed disturbance rates across the 77 landscapes, showing that the prevailing disturbance regime and disturbance agents are of considerable importance for the local manifestation of disturbances at the landscape scale. An additional 32% of the variability was explained by the random variation in disturbance rates among landscapes and years, highlighting that despite their dependence on the prevailing disturbance agents, disturbance events can be highly stochastic both in space and time. In total, our model explained 57% of the variability in observed disturbance rates across the temperate and boreal biome.

**TABLE 2:** Summary of the annual rates of natural stand‐replacing disturbances derived from remote sensing data (Hansen et al., 2013) in 77 strictly‐protected landscapes.

Cluster	Disturbance rate (% yr^−1^)					Number of landscapes
	Mean	Median	Standard deviation	Minimum	Maximum	
Low	0.03	0.01	0.07	0.00	3.58	18
Moderate	0.08	0.03	0.19	0.00	6.93	34
High	0.84	0.32	1.78	0.00	42.52	25

*Note*: Disturbance rates were calculated for three clusters of disturbance activity following definitions given in Seidl et al. (2020) and Sommerfeld et al. (2018).

The disturbance activity clusters, and thus the large‐scale variation in disturbance rates, could be successfully modelled as a function of the community mean wood density and the 30‐year mean annual temperature range of each landscape (Figure 1; Table S1). The disturbance activity clusters thus provide a parsimonious link between our empirical disturbance model (Table 2) and the output of dynamic vegetation models (i.e. simulated forest type combined with climate). The model could attribute the correct disturbance activity cluster (and thus the corresponding disturbance rate) with an average accuracy of 55% and was significantly better than a null‐model with random cluster assignment (likelihood ratio test: $\chi^{2}$(3) = 34.75, *p* < 0.01). The model was very good at distinguishing between high and low activity clusters, with no misattributions between these two classes (Table S2). Overall, the high disturbance activity cluster was attributed with high confidence (only 24% of high disturbance activity landscapes were attributed as moderate), but there was lower confidence in distinguishing between the low and moderate disturbance activity clusters (Table S2). In general, we found that the probability of belonging to the high disturbance activity cluster increased with decreasing wood density and greater annual temperature ranges (Figure 1e), whereas areas of low temperature range and high wood density have a higher probability of being characterized by low disturbance activity (Figure 1e). The moderate disturbance activity cluster lies in between the two end members; that is, it is characterized by average wood density and temperature range. Calculating weighted average disturbance rates based on the probability of belonging to a disturbance cluster then allowed to link temperature and wood density directly to disturbance rate. This process introduced no notable bias in the overall disturbance rate, with the mean disturbance rate across the 77 landscapes being 0.29% yr^−1^ whether calculated directly from the observations or via our cluster‐based model.

These results are consistent with our understanding of the major disturbance agents and tree traits in global temperate and boreal forests. For instance, it is well established that dense wood tends to be an indicator of an ecological strategy that favours persistence (e.g. defence against biotic agents and fire) over maximizing productivity (Stephenson et al., 2011). Moreover, the trees that are susceptible to mortality during large bark beetle outbreaks are conifers, which have relatively low wood density (Hicke et al., 2012); and large annual temperature ranges are characteristic of a continental climate with hot summers, which may predispose ecosystems towards large fires. The predictors of conifer share and maximum tree height were also tested based on mechanistic reasoning, as they have previously been found to be correlated with disturbance activity in temperate forests (Sommerfeld et al., 2018), but they did not improve the discriminating power between disturbance activity clusters (Table S1) and were thus not included in the final model.

### 3.2 Disturbance return intervals across the temperate and boreal biomes

**FIGURE 3:**
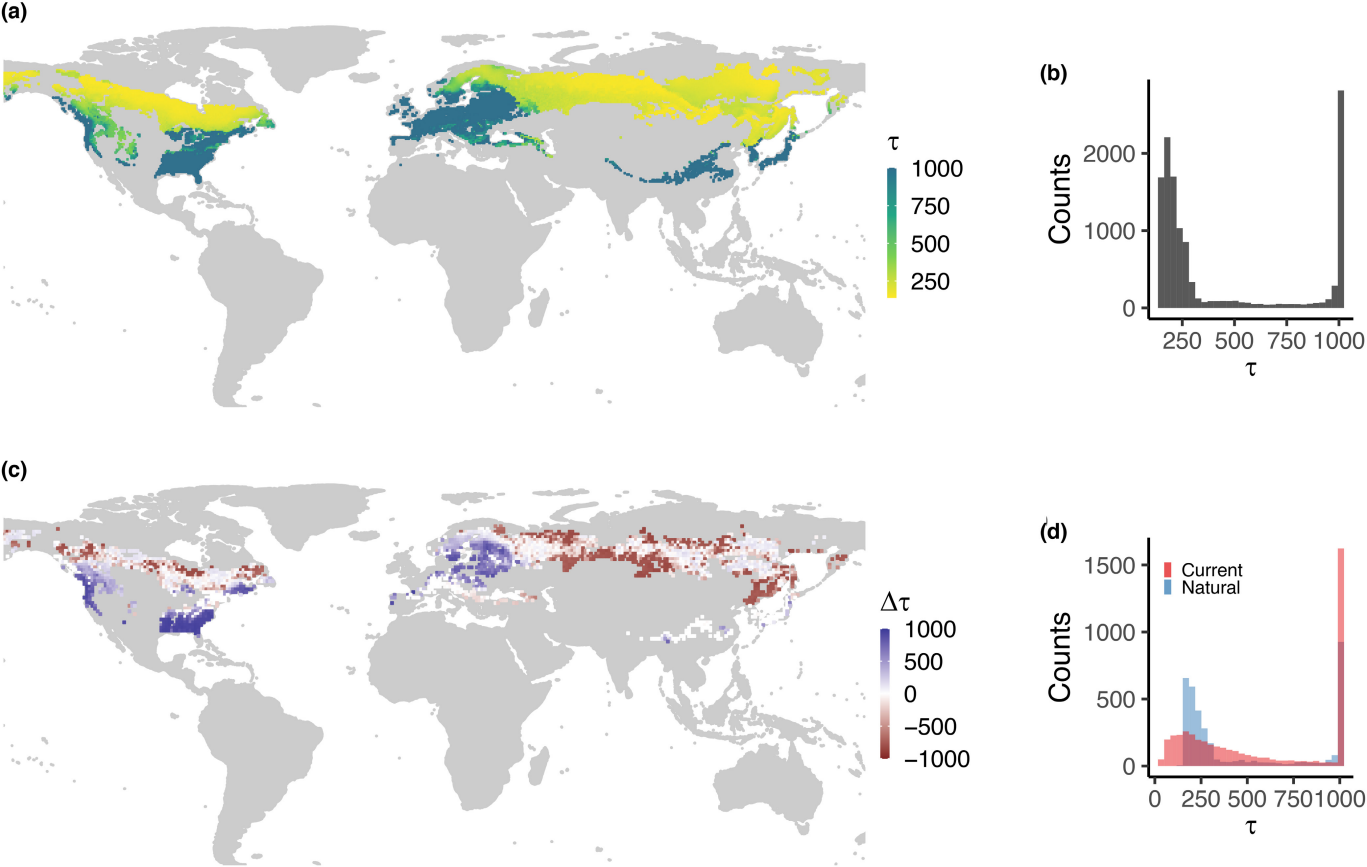
Stand‐replacing disturbance return intervals in natural forest and compared with current forest. (a) Natural forest disturbance return interval (years) as calculated using LPJ‐GUESS employing the empirical disturbance modelling approach developed here. Return intervals were capped at 1000 years. (b) Frequency distribution of disturbance return interval. Each count represents one 0.5° × 0.5° grid cell. (c) Difference between stand‐replacing disturbance return intervals in natural forest and in current forest (Pugh, Arneth, et al., 2019). Positive values indicate a disturbance surplus in current forests compared with natural conditions, whilst negative values suggest a current disturbance deficit. Results are calculated and shown based on closed‐canopy forest for consistency with Pugh, Arneth, et al. (2019). (d) Difference in frequency distribution between natural and current forests. For areas outside the observed trait or climate space, see Figure S3.

Scaling the results from the 77 landscapes across the temperate and boreal forest biomes, using LPJ‐GUESS, identified a clear gradient in typical disturbance return interval in unmanaged forests, where return interval is here approximated by disturbance rotation time, that is the reciprocal of the disturbance rate. Disturbance return interval had a median of 204 years in boreal forest and greater than 1000 years in temperate forests (Figure 3a). The frequency distribution of return interval showed a strong left‐skew, representing areas typically experiencing stand‐replacing disturbance at least once every 400 years, that is boreal needleleaf and mixed forests, with a long tail towards rare disturbances across temperate broadleaf forests (Figure 3b).

Much of the range of temperate and boreal forests falls within the trait space (as calculated based on LPJ‐GUESS vegetation composition) and climate space of the 77 landscapes; however, some parts of particularly western Europe and far‐eastern Siberia do not, leading to reduced confidence in the simulated disturbance rates in these regions (Figure S3). Standard error estimates of disturbance rate were generally low, but reached up to 30% in southern boreal forests (Figure S4), where a relatively small difference in disturbance rate could substantially alter the abundance of early versus late successional PFTs. There are limited direct observations of disturbance rates in unmanaged forest; however, our results are qualitatively consistent with evidence of current fire regimes in boreal forests (De Groot et al., 2013; Pugh, Arneth, et al., 2019; White et al., 2017) and an apparent rarity of major stand‐replacing disturbance events in many temperate forests (Frelich & Lorimer, 1991; Janda et al., 2017; Lorimer, 1989; Pickett & White, 1985; Thom et al., 2013).

In regions where the disturbance regime is dominated by fire and in which fires tend towards being stand‐replacing, fire return intervals derived from charcoal records in lake sediments or dendrological fire scars can provide a cross‐check on our results. Such observations do not provide a like‐for‐like comparison with our estimates for several reasons. First, they relate to periods which generally had a different mean climate and, in the case of charcoal records, a different lag relative to the last ice age (which has consequent impacts on the extent to which northward species migration has occurred, e.g. Higuera et al., 2009). Second, they may include the impacts of megafauna, whose effects we cannot assess with our method, given that in the modern world these have been lost or their abundance greatly reduced. Nonetheless, these comparisons are useful for assessing the general magnitude of disturbance intervals simulated here. We compared against eight such sites in the boreal zone (Table S4). In four cases, our stand‐replacing disturbance return intervals were within the confidence limits of the fire return intervals, and in four cases, they were 1.6 to 2.4 times longer (i.e. simulating fewer disturbances than observed). Given that not all observed fires may have been stand replacing, whilst we here only simulate stand‐replacing disturbances, a bias of this order is within reasonable expectations. Overall, we conclude that the various lines of evidence in the literature are consistent with the pattern of disturbances generated by our simulations.

The distribution of simulated disturbance return intervals for unmanaged forests differs markedly in many regions from that of recent disturbance rates observed in closed‐canopy forests (Pugh, Arneth, et al., 2019). The vast majority of temperate forests in Europe, Japan, U.S.A. and western Canada show a substantial disturbance surplus, with return intervals reduced by several hundred years or more through anthropogenic influence (Figure 3c; unmanaged rates adjusted for closed‐canopy forests for consistency, see Methods). Exceptions are central Europe, where clear‐cut forestry is increasingly rare (Duncker et al., 2012), and the north‐eastern U.S.A., where many forests are recovering from historical land‐use change (Pan et al., 2011). The disturbance surplus is particularly marked in the south‐eastern U.S.A., which would naturally be expected to include a mixture of broadleaf and conifer species (Figure S5; Hengl et al., 2018), but is today an area of intensive plantation forestry, concentrating on needleleaf species (Figure S6). The surplus in this latter area may be slightly overstated, as LPJ‐GUESS tends to simulate this region as pure broadleaf under natural disturbances only, which decreases disturbance rates based on the higher average wood density (Figure 1f). In contrast, return intervals in much of the boreal are similar for both present‐day and unmanaged forests (Figure 3c). This may reflect the lack of compositional change in these forests combined with the primacy of large fire disturbances. However, some of the areas where return intervals are similar are also heavily managed, for example southern Canadian forests (Curtis et al., 2018), perhaps indicating that harvesting successfully emulates natural disturbance regimes in these areas.

Boreal forests also include large regions of disturbance deficit, which are particularly marked in Southern Siberia and North‐Eastern China. These are areas that are currently dominated by broadleaved birch and aspen forests (Schepaschenko et al., 2011; Figure S6) and where the primary form of disturbance is forestry (Curtis et al., 2018). Our simulations suggest that these areas would be mixed broadleaf‐needleleaf forests without the influence of forestry or land‐use legacies (Figure S6). Maps of potential natural vegetation derived from historical and palaeo‐observations (Haxeltine & Prentice, 1996; Hengl et al., 2018; Figure S5) tend to suggest an even stronger component of needleleaf trees, which would even further accelerate disturbance rates in our model, exacerbating the deficit. A plausible explanation of the apparent disturbance deficit in these forests is that past fire activity accelerated by human influence (Mollicone et al., 2006) has pushed them towards their early successional states, which are less prone to stand‐replacing fire events (Johnstone et al., 2011). For the other areas of disturbance deficit scattered across the northern boreal forest, the drivers are less clear and it is of course possible that limitations in our approach play a role (see Section 3.4).

### 3.3 Impacts on forest structure and carbon turnover

**FIGURE 4:**
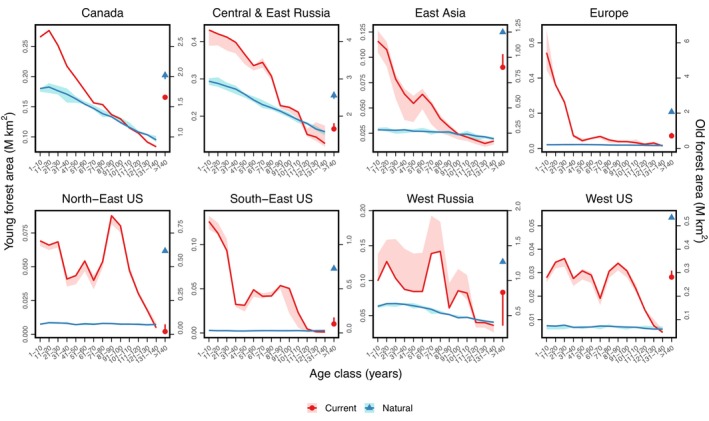
Stand age distributions for forest assuming equilibrium with natural forest disturbance rates (blue lines) and taking into account the historical evolution of anthropogenic land‐use change and forest harvest (red lines) for eight regions across northern hemisphere temperate and boreal forests, as calculated by the LPJ‐GUESS vegetation model. Total forest area for stand age classes less than 140 years old (lines) are displayed relative to the left‐hand axis, whilst those for old (>140 years) forest stands (symbols, positioned rightmost in each panel) are shown relative to the right‐hand axis. The blue shaded region shows the effect of ± 2 standard errors in the natural disturbance rate estimates applied in the LPJ‐GUESS model. The red shaded region shows the effect of using the upper or lower estimates of land‐use change and forest harvest supplied with the LUH2 dataset. In the case of old forest (symbols), the uncertainties are indicated by error bars.

What does the human‐induced shift in disturbance rates mean for forest age structure and function? The anthropogenic influence on forest age structure is pervasive in all biomes, resulting in a younger forest (Figure 4). This shift is more limited in boreal forests, reflecting the similarities in disturbance return interval (Figure 4). However, the large anthropogenic changes in disturbance rate lead to very different age structures across temperate forests (Figure 4). Overall, current temperate and boreal forests are estimated to have 44% (5.00 million km^2^) less old forest (stands more than 140 years old) compared with their state subject to only natural disturbances, with the losses concentrated in the eastern USA and Europe (Figure S7).

These estimates of changes in age structure should not be interpreted as precise reconstructions, but rather to be indicative of the level and character of change. This caveat arises because, although we account for the important role of land‐use history in realized age structure (Caspersen et al., 2000; Hurtt et al., 2020; Pan et al., 2011), the age structures calculated here are based on the assumption of homogeneity at a scale of 0.5° × 0.5°. In reality, the complexity of landscapes that are found at subgrid‐cell scale may result in more diverse age structures than those reconstructed here, although it is also plausible that any differences may cancel with upscaling. Similarly, tree age structure at sub‐stand level driven by non‐stand‐replacing disturbances is also not accounted for here. Nonetheless, the calculations herein demonstrate major demographic shifts in temperate forests due to human land use, consistent with theoretical expectations (McDowell et al., 2020). The present‐day age structure estimates show consistency with an independent forest inventory‐based estimate for several regions (Figure S8). Using this inventory‐based estimate instead as the present‐day basis for comparison would give a reduction in old forest stands of 56% (6.43 million km^2^), yielding the same conclusion as using the LUH2 dataset.

Younger, more regularly disturbed forests imply lower carbon stocks and a shorter vegetation carbon turnover time. Total vegetation carbon stocks on existing forest area were reduced by 30.0% in the temperate and 6.8% in the boreal zone (Table 3). The total vegetation carbon turnover time for temperate forests was reduced by 32.1% (10th and 90th percentiles of change at grid‐cell level, −71.6% and 1.9%) in our simulations due to anthropogenic disturbances. For boreal forests, the corresponding reduction was 7.1% (−21.3%, 11.1%). Much of this enhanced turnover will be directly removed from the ecosystem in the form of harvest, reducing carbon turnover time in the forest ecosystem overall, although how quickly harvested carbon is returned to the atmosphere is dependent on the fate of the wood products produced (Mason Earles et al., 2012). The removal of material from the ecosystem, combined with smaller live carbon stocks, means that more frequent disturbance does not equate to more litter (here defined as the sum of deadwood and dead soft tissues) in the forest. In our simulations, litter stocks in temperate zone forests were reduced by 17.5% (−61.6%, 9.8%; Table 3), equating to a substantial reduction in habitats for insects and microorganisms (Sandström et al., 2019).

**TABLE 3:** Impact of disturbance changes on forest carbon cycling.

		Natural disturbance	With forestry and land‐use legacies	Change (%)
Temperate	Vegetation C stock (Pg)	74.5	52.1	−30
	Litter C stock (Pg)	61.2	50.5	−17
	Soil C stock (Pg)	135.8	133.0	−2
	Vegetation C turnover time (yr)	16.6	11.3	−32
	Ecosystem C turnover time (yr)	60.6	51.0	−16
Boreal	Vegetation C stock (Pg)	60.5	56.4	−7
	Litter C stock (Pg)	108.6	103.9	−4
	Soil C stock (Pg)	364.1	361.7	−1
	Vegetation C turnover time (yr)	8.9	8.3	−7
	Ecosystem C turnover time (yr)	78.7	76.8	−2

### 3.4 Limitations of the approach

Despite efforts to use state‐of‐the‐art disturbance products and modelling tools available at this scale and to evaluate and cross‐check with complementary sources of information, quantifying the impact of forestry and land‐use change legacies on temperate and boreal forests remains an underconstrained problem. Protected forests are, of course, not entirely without human influence, some of these forests were originally planted or have a history of fire suppression. There may also be biases in their designation as protected, with historically less disturbed landscapes perhaps more likely to be designated as worthy of protection, analogous to the hypothesized majestic forest bias (Malhi et al., 2002). These effects have the potential to bias disturbance rates in either direction relative to the true natural values. But despite these limitations, these protected forests offer the best available window into natural forest dynamics under recent climate conditions.

Possible further sources of bias include that (**a**) disturbance return intervals are overestimated due to missing infrequent large disturbance events, or conversely (**b**) that disturbance rates are overestimated because several landscapes are impacted simultaneously by the same large weather anomaly, that (**c**) our disturbance algorithm may overestimate the influence of climate in these marginal regions, or (**d**) the vegetation simulated by LPJ‐GUESS (Figure S5) may differ from that which would occur in reality. None of these can be ruled out because of the lack of clear past analogues for 20th–21st century climate conditions and because of human influences beside forestry (e.g. megafauna extinction and fire suppression). Indeed, **a**, **c** and **d** are all potential explanations for the areas of disturbance deficit identified in Figure 3c. Nonetheless, the wide geographic spread of the protected landscapes (Seidl et al., 2020), along with their large total forest area of 15,440,181 ha, both act to mitigate against eventualities **a** and **b**. Furthermore, LPJ‐GUESS performs well in reproducing the key features of forest succession across the boreal zone, which is the key building block for an appropriate reconstruction of vegetation composition (Supplementary Information Section S1, Figure S1), thus mitigating against eventuality **d**.

We used here a 14‐year observational period, as this is the longest consistently calculated time series currently available across all landscapes (Palahí et al., 2021), but as more of the Landsat archive is utilized in large‐scale disturbance products, such as now available for Europe (Senf & Seidl, 2021a), time series of 40 years or more may become possible in the future. Furthermore, the emerging ability to attribute disturbance agents in such products (Kennedy et al., 2015; Senf & Seidl, 2021b) will open up the possibility of generating simple agent‐specific models for stand‐replacing disturbance events, such as already exist for burnt area (Knorr et al., 2016), allowing to more accurately assess the fate of carbon following tree death (i.e. whether carbon is burnt, falls to the ground, or remains standing in snags), and thus improve the fidelity of carbon residence time estimates.

When it comes to converting age structures and disturbance rates into estimates of biomass and carbon turnover, LPJ‐GUESS has a strong pedigree, having been extensively evaluated for these aspects (Lindeskog et al., 2021; Pugh, Arneth, et al., 2019; Pugh, Lindeskog, et al., 2019; Smith et al., 2014) Furthermore, our evaluation of biomass stocks in the protected areas revealed strong consistency between model and observations (Figure S9), with an *R* ^2^ of 0.42 and a mean bias of −0.26 kg C m^−2^ (compared with a mean observed biomass across all landscapes of 7.26 kg C m^−2^). Our overall estimates of vegetation biomass including forestry and land‐use legacies are consistent with independent estimates for the temperate forest of 25–53.2 Pg C (Thurner et al., 2014) and 32–64 Pg C (Erb et al., 2018) and for the boreal forest of 30–56.4 Pg C (Thurner et al., 2014) and 35–64 Pg C (Erb et al., 2018).

Total ecosystem carbon turnover time has previously been estimated using gross primary productivity (GPP) instead of NPP by Carvalhais et al. (2014)), who estimated 95% confidence intervals of 18.9–30.8 years for the temperate biome and 45.4–73.4 years for the boreal biome. Replacing NPP with GPP in Equation 1 and taking simulation LUHPmid (Table 1) for maximum consistency, our independent estimates of 19.9 years for the temperate biome and 40.8 years for the boreal biome sit on the low end of their ranges. For vegetation carbon turnover time, our estimates for temperate forest are very close to those reported by Erb et al., 2016), who estimated 11.0 years for current vegetation and 16.7 years in the absence of human actions. For boreal forests, the equivalent estimates were 15.3 and 19.5 years, respectively, which is approximately double the values in our simulations. Erb et al. do not report uncertainties on these values, but given the underlying NPP and biomass values used differ by a factor of more than two for the boreal forest (Erb et al., 2016, table S16), our estimates do not appear to be inconsistent. Overall, whilst absolute turnover time estimates remain quite uncertain, the conclusions based on the relative changes in turnover time which we report are likely to be robust to any possible biases in our estimates.

Our objectives in this study were to determine the effects of human land use on the forest disturbance regime; however, human activity also alters disturbance regimes through changes in the climate system. We did not address such climate‐driven changes in disturbance rates here, although they may be becoming increasingly important drivers of forest dynamics (McDowell et al., 2020). Relationships of interannual climate variability with disturbance rates have previously been identified (Sommerfeld et al., 2018), but these relationships have limited power in long‐term extrapolation across changing climate normals because they are based on the anomaly of weather in a given year to the climate normal. That is, given one year of anomalous weather, anomalous disturbance rates might be expected, but given many years of such conditions, the coupled vegetation‐disturbance system will shift to a new state and dynamic, invalidating links between weather and disturbance rates that were based on the old state and dynamic. Despite the uncertainties, process‐based models remain the best tools to extrapolate to novel conditions (Seidl et al., 2011).

## 4 CONCLUSIONS

The semiempirical natural disturbance modelling approach for estimating disturbance rates in the absence of human influence, working from emergent outcomes, but grounded in process‐based knowledge, offers a middle way between subsuming natural disturbances within a generic mortality rate and applying complex disturbance models that are difficult to constrain across large scales (e.g. Jönsson et al., 2012; Thonicke et al., 2010). It thus provides a lightweight and data‐driven modelling approach for the inclusion of natural disturbances in large‐scale demographic vegetation models and Earth system models, particularly for the historical period. This algorithm provides the complement to existing information for land‐use change and wood harvest (Hurtt et al., 2020), making it possible to realistically simulate forest age structure and thus its implications for ecosystem function, from carbon uptake to biodiversity (Fisher et al., 2018).

That humans, through forestry and land‐use legacies, have increased forest disturbance rates relative to their natural state, thereby reducing forest age and increasing carbon turnover, has long been assumed, but has eluded quantification at the scale of biomes. We have provided here a quantitative northern‐hemisphere‐scale assessment of human impact on forest disturbance regimes and the downstream effect of these changes on forest age structure and carbon turnover. The consistency of the results herein with observations in relatively undeveloped parts of the boreal (Pugh, Arneth, et al., 2019) supports the credibility of the simulations, although results for eastern Siberia must be taken with considerable caution due to a lack of reference landscapes underlying the disturbance model in this region (Figures 1a and S3). Our results provide a context against which to assess the extent to which human actions have changed the dynamics, state and function of boreal and temperate forests. They likewise illustrate the potential change that might result from a shift towards unmanaged forest as part of a Nature‐based solutions approach (Wild et al., 2020). Considering the impact of management on disturbance regimes should be a core aspect of any such policies.

## CONFLICT OF INTEREST STATEMENT

The authors declare no conflict of interest.

## BIOSKETCHES

**Dr. Thomas Pugh** is associate professor at Lund University, Sweden, and Reader in Biosphere‐Atmosphere Exchange at the University of Birmingham, U.K. His work uses modelling and observation‐based approaches to explore interactions between the terrestrial biosphere, climate and management at the global scale, with a focus on forest dynamics.

**Dr. Cornelius Senf** is a research scientist at the Ecosystem Dynamics and Forest Management Group of Technical University of Munich, Germany. His research focusses on understanding the dynamics of forest ecosystems using remote sensing approaches, with a particular focus on Europe.

## Supporting information


**Data S1:** Supporting Information

Table S5.

## Data Availability

Model simulation results are archived on Zenodo under 10.5281/zenodo.5590735, as are the underlying LPJ–GUESS model code and simulation settings under 10.5281/zenodo.8419062. All code underlying the analyses is available from https://github.com/pughtam/NatDist, 10.5281/zenodo.8419183. New users of the LPJ–GUESS model are recommended to access the latest release and support via http://web.nateko.lu.se/lpj‐guess/.
