# Supplementary material for: The anthropogenic imprint on temperate and boreal forest demography and carbon turnover
Source: Glob Ecol Biogeogr. 2023 Oct 16;33(1):100–15. doi: 10.1111/geb.13773 (PMC10952773; doi:10.1111/geb.13773)
Supplement: Supplementary file 1 — Data S1: Supporting Information [file GEB-33-100-s001.pdf]

Supplementary information for:

The anthropogenic imprint on temperate and boreal forest demography and carbon turnover

Thomas A. M. Pugh[1,2,3*], Rupert Seidl[4,5], Daijun Liu[2,3,6], Mats Lindeskog[1], Louise P. Chini[7], Cornelius Senf[4*]

[1] Department of Physical Geography and Ecosystem Science, Lund University, Sweden.
[2] School of Geography, Earth and Environmental Science, University of Birmingham, Birmingham, UK.
[3] Birmingham Institute of Forest Research, University of Birmingham, Birmingham, UK.
[4] Ecosystem dynamics and forest management group, Technical University of Munich, Freising, Germany.
[5] Berchtesgaden National Park, Berchtesgaden, Germany.
[6] Department of Botany and Biodiversity Research, University of Vienna, Rennweg 14, 1030 Vienna, Austria.
[7] Department of Geographical Sciences, University of Maryland, College Park, MD 20742, USA.

* Correspondence to: Thomas A. M. Pugh, thomas.pugh@nateko.lu.se

S1. Evaluation of successional trajectories in the boreal forest.

Given the importance of the relative importance of composition of conifers vs broadleaved species in our disturbance simulations (as mediated by differences in wood density), we compared LPJ-GUESS successional sequences throughout the boreal zone with those reported in the literature for nine locations (Figure S1). All LPJ-GUESS simulations were based on recovery from bare ground under a constant climate and CO_2 forcing, as used during the model spin-up period (see main text, Methods). In making this comparison it is important to consider that LPJ-GUESS simulations are characterizing a landscape, with many replicate patches which may follow different trajectories.

1. In the Russian boreal forests in the Arkhangelsk region (simulated grid cell at 44.25°E, 61.75°N), *Betula* species (IBS) typically establish initially, giving way to *Picea abies* (BNE) after ca. 80 years (Shorohova et al., 2009). Simulations from LPJ-GUESS show the same character, although BNE dominance is delayed ca. 20-30 years compared to the observations (Fig. S1a).

2. In northern Finland (26.75°E, 66.75°N), *Betula* species (IBS) dominate initially, with dominance transitioning to *Picea abies* (BNE) after about 80 years (Shorohova et al., 2009). Whilst LPJ-GUESS simulates the stand transition to *Picea* dominance at about the right time, it simulates BINE, whose equivalent in this system is *Pinus sylvestris*, as the early successional species, with only a minor role of IBS (Fig. S1b).

3. In the southern boreal zone of Russia (71.25°E, 56.25°N), deciduous species dominate for ca. 100 years, before *Picea* and *Abies* (BNE) gain dominance (Shorohova et al., 2009) with *Pinus siberica* (BINE) coming in after ca. 180 years. LPJ-GUESS simulates the transition from deciduous to late-successional conifers well (Fig. S1c). The later transition to *Pinus siberica* is not captured, but LPJ-GUESS does not include a mechanism to facilitate this kind of transition to a less shade-tolerant species without restarting succession. The very minor difference in wood density between *Pinus* and *Abies* species (Fig. S2) mean that this is not consequential for our disturbance results however.

4. In the northern boreal zone of Russia (67.25°E, 62.75°N), *Betula* species (IBS) dominate initially, transitioning to dominance of *Pinus sibericus* and *Pinus sylvestris* after 60 to 80 years (Shorohova et al., 2009). Here, LPJ-GUESS captures well the demise of the IBS, but, whilst a substantial *Pinus* component exists, tends towards *Picea* dominance (Fig. S1d). As in the southern zone, however, the similarity in wood density means there is little consequence for our disturbance results.

5. In the forests around Lac Duparquet in eastern Canada (79.25°W, 48.25°N) successional cycles usually follow *Populus tremuloides* and *Betula papyrifera* (i.e. IBS) dominating initially, being replaced by *Abies balsamea* (BNE) after about 150 years (Bergeron & Fenton, 2012). LPJ-GUESS shows a somewhat quicker decline of IBS than the observations, but is broadly consistent with the point of switching to BNE dominance (Fig. S1e).

6. The lowland forests around Lake Matagami in eastern Canada (78.25°W, 49.25°N) may find *Picea mariana* (BNE) dominating in both early and late regrowth periods, or successional changes after about 100 years from *Pinus banksiana* (BINE), *Populus tremuloides* (IBS) or *Betula papyrifera* (IBS) to *Picea mariana* or to a mix of *Picea mariana* and *Pinus banksiana* (Lecomte & Bergeron, 2005). LPJ-GUESS shows a high level of consistency with these observations, with all three types PFTs present at substantial levels during early succession and *Pinus* persisting into late succession (Fig. S1f). Probably the abundance of BNE is underestimated in early succession.

7. Forests in the basin of the Tanana River in Alaska (148.75°W, 64.75°N) follow a trajectory of either *Populus tremuloides* or *Betula papyrifera* (IBS) being replaced by *Picea glauca* (BINE) after ca. 100

years, typical of the upland sites, or *Populus balsamifera* (IBS) being replaced by *Picea glauca* after ca. 125 years (typical of the floodplain sites) (van Cleve & Viereck, 1981). LPJ-GUESS simulates well the transition point between the early and late successional, but simulates a BNE, rather than a BINE as the climax species (Fig. S1g). *Picea glauca*, however, is clearly shade tolerant relative to the early successional species here and sources are inconsistent on its overall classification (Marfo & Dang, 2009; Niinemets & Valladares, 2006), so this difference likely results more from the coarse level of classification used, than a substantial error in the simulated forest.

8. An assessment of 53 stands in central Ontario, Canada (89.25°W, 49.25°N) found that after an initial burst of *Populus tremuloides* and *Betula papyrefera* (IBS) dominating for about 30 years, coniferous species tended to become at least co-dominant, with *Pinus banksiana* (BINE) giving way to *Picea mariana* or *Abies balsamea* (BNE) over the period of 150-200 years after the beginning of succession (Taylor et al., 2020). LPJ-GUESS captures the transition from BINE to BNE well, but tends to underestimate the amount of IBS in the landscape (Fig. S1h), which may suggest that non-stand-replacing disturbances are being underestimated in the model.

9. In the northern Rocky Mountains, Canada (123.75°W, 58.25°N), *Pinus contorta* (BINE) is observed to dominate the early stages of succession, giving way to *Picea engelmannii* (BNE) after about 150 years (Heinselman, 1981). This character is well captured by LPJ-GUESS (Fig. S1i).

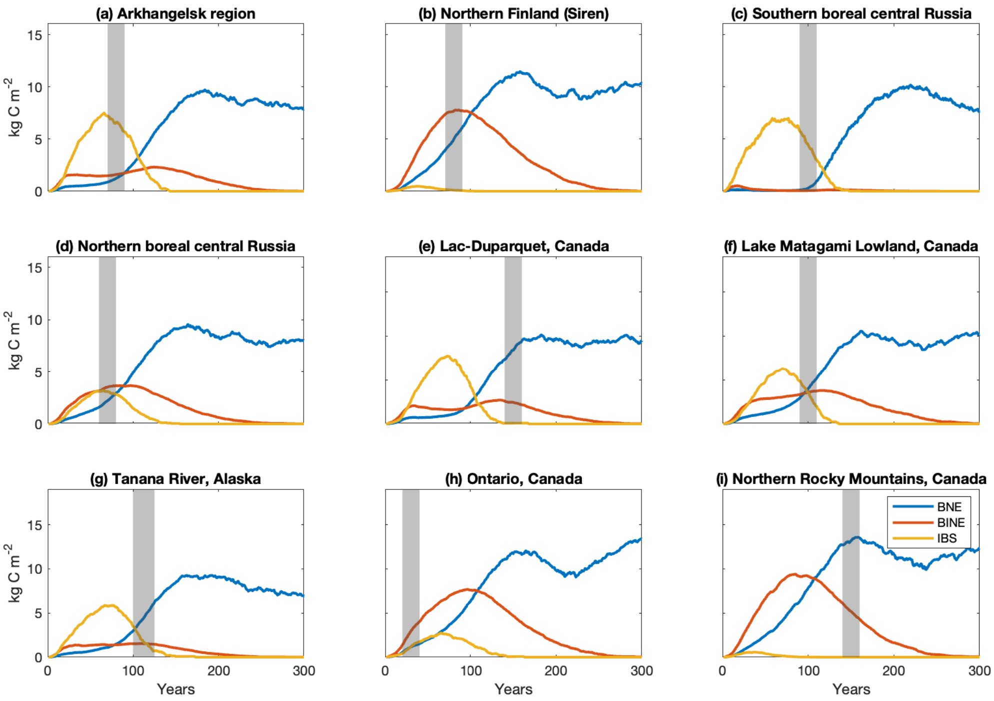

Figure S1. Successional trajectories of total vegetation biomass simulated by LPJ-GUESS for nine regions across the boreal forest. Colours show the contributions for different plant functional types (BNE = Boreal needleleaf evergreen shade-tolerant tree, BINE = Boreal needleleaf evergreen shade-intolerant tree, IBS = temperate/boreal shade-intolerant summergreen tree). Shaded areas indicate the point of the observed transition of composition dominance, as described in the text in Section S1.

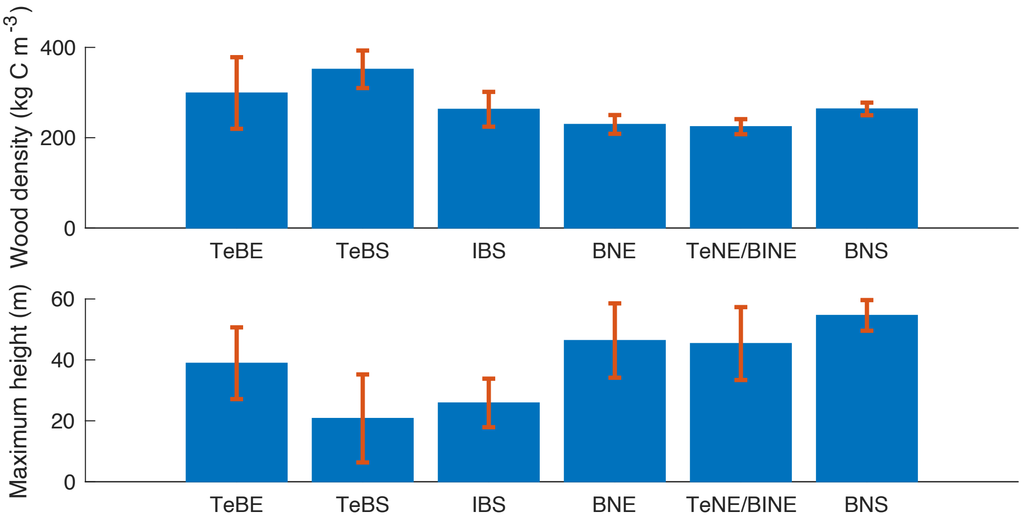

Figure S2. Trait values assigned to each of the LPJ-GUESS plant functional types based on means of the species-level values weighted by their abundance across the landscapes. Error bars show ±1 standard deviation.

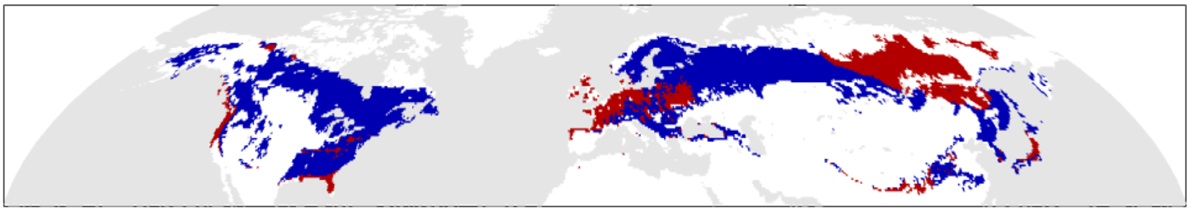

Figure S3. Areas which are outside the wood density or temperate range space of the 77 reference landscapes (red) and inside (blue).

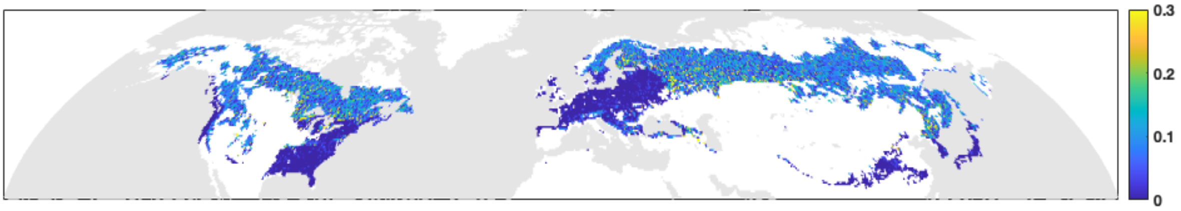

Figure S4. The 95% confidence interval of the disturbance return interval estimates from LPJ-GUESS, presented as an absolute fraction of the best estimate. Calculated as $(\tau_{NatPhigh}-\tau_{NatPlow})/\tau_{NatPmid}$. Areas of no difference result because all estimates of return intervals exceed the limit of 1000 years. The speckling effect is a result of the stochasticity of the model being accentuated when differences between two simulations are taken.

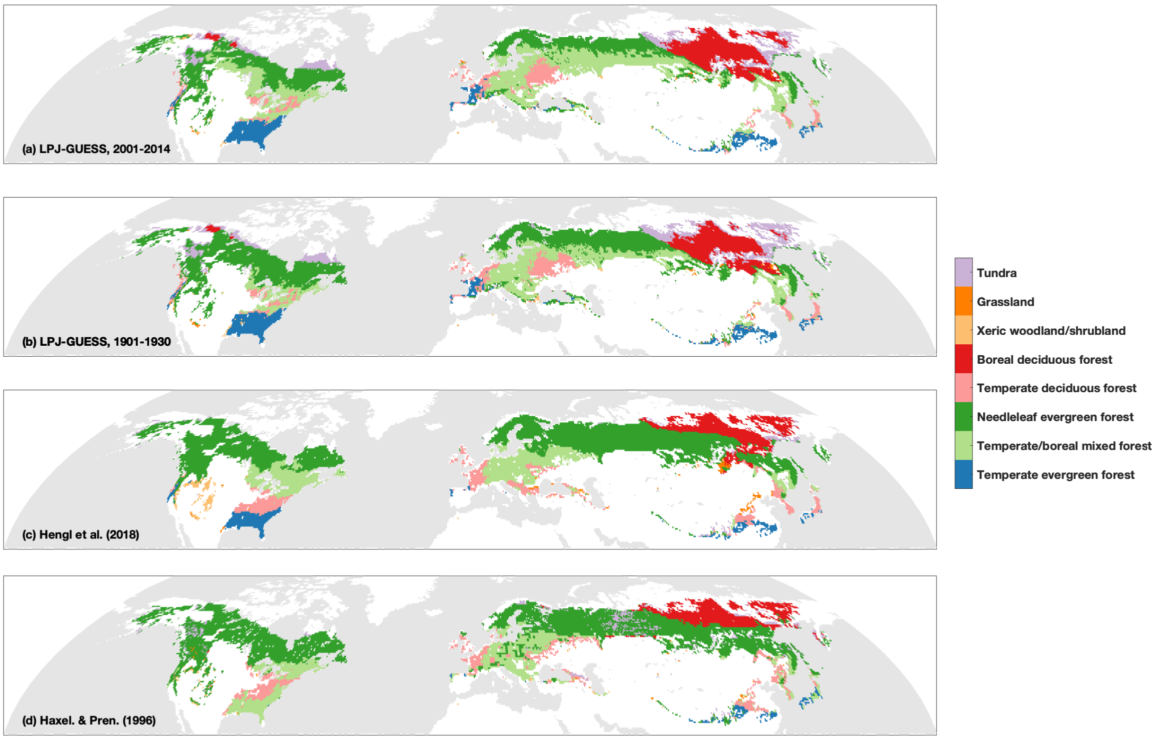

Figure S5. LPJ-GUESS biome distribution from the TCmid simulation for the recent historical and early 20th century time periods and those from two independent estimates of potential natural vegetation distribution (Haxeltine & Prentice, 1996; Hengl et al., 2018). The early 20th century LPJ-GUESS distribution is included as a comparator without substantial anthropogenic climate change and atmospheric composition signals and to demonstrate the difference that these signals have on vegetation distribution.

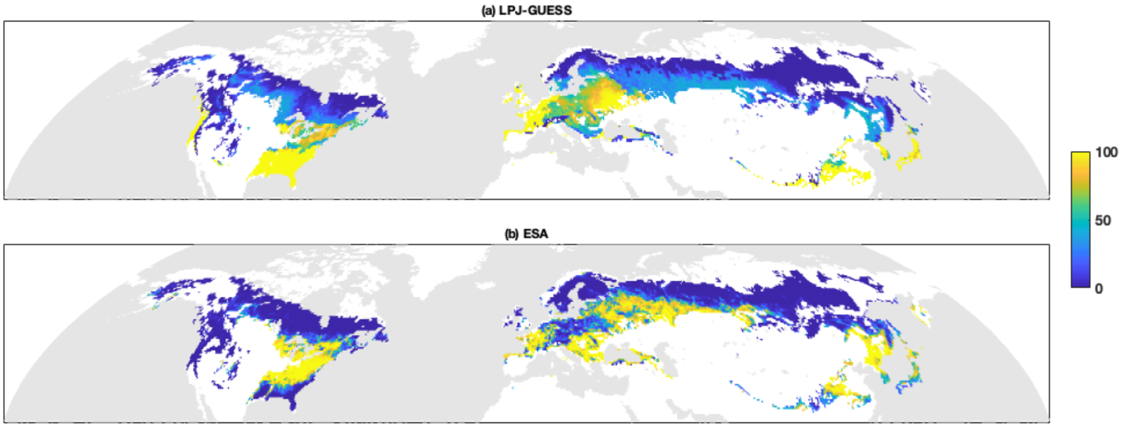

Figure S6. Percentage of tree cover as broadleaf, based on LPJ-GUESS (top) and ESA CCI (bottom).

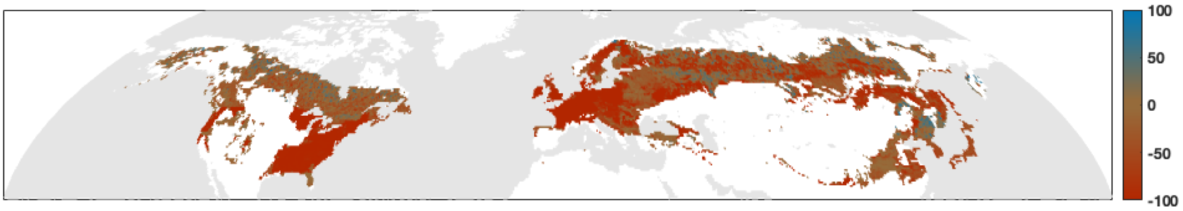

Figure S7. Percentage difference in old (>140 years) forest area in the year 2014 between simulations with only natural disturbances and simulations included both natural and human-induced disturbances. Red colours indicate less old forest when human-induced disturbances are considered, whilst blue indicates more old forest and areas of limited change are shown in brown.

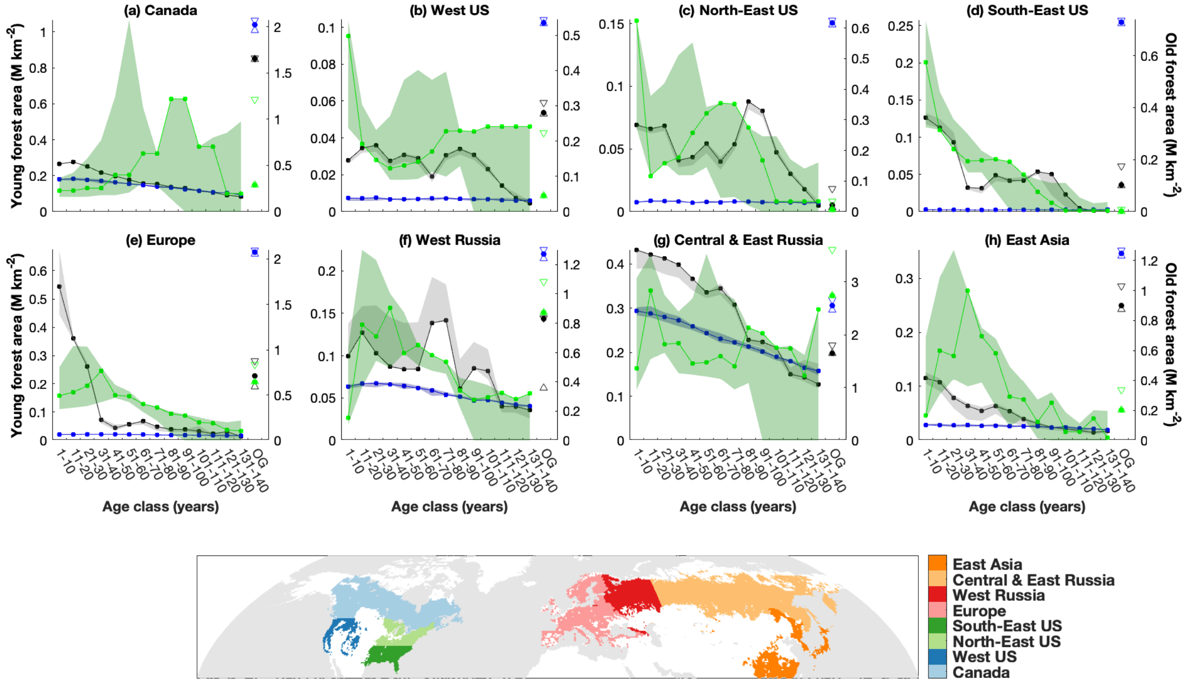

Figure S8. Stand age distributions for forest (as in Fig. 4) assuming equilibrium with natural forest disturbance rates (blue lines) and taking into account anthropogenic land-use change and forest harvest (black lines) for eight regions across northern hemisphere temperate and boreal forests, as calculated by the LPJ-GUESS vegetation model. Green lines show age structure estimates from the Global Forest Age Dataset v1.1 (Poulter et al., 2019; Pugh et al., 2019) GFAD age distributions are rescaled to the LUH2 forest area to give a like-for-like comparison. Total forest area for stand age classes less than 140 years old are displayed relative to the left-hand axis, whilst those for old forest are shown relative to the right right-hand axis. The blue shaded region shows the effect of ±2 standard errors in the natural disturbance rate estimates applied in the LPJ-GUESS model. The grey shaded region shows the effect of using the upper or lower estimates of land-use change and forest harvest supplied with the LUH2 dataset. Green shading shows the 95% confidence limits of GFAD. In the case of old forest, the uncertainties are indicated by triangles.

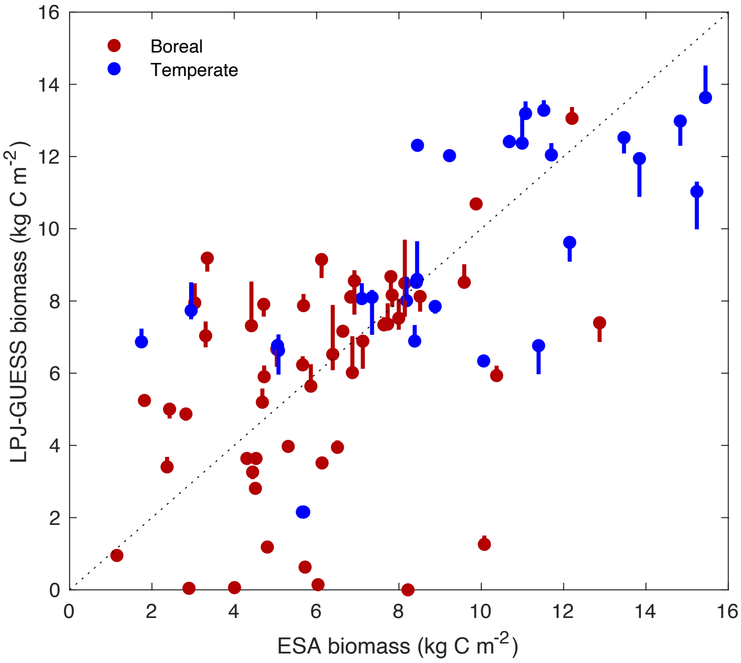

Figure S9. Evaluation of LPJ-GUESS simulation biomass stocks for the 77 protected landscapes, based on the simulation with natural disturbances only, against ESA Biomass CCI. Vertical lines indicate the range of biomass resulting from the NatPmid, NatPlow and NatPhigh simulations. Note that maximum and minimum rates of disturbance do not necessarily translate into minimum and maximum biomass, due to the non-linearities induced by the interaction of disturbance rate and vegetation composition.

Table S1: Comparison of the different multinomial models linking disturbance clusters to climate and traits. Models were compared using the Akaike Information Criterion (AIC) and the model with the lowest AIC was taken as final model.

**Model**	**Predictors**							**AIC**
	**Wood density**	**Max. height**	**Share of conifers**	**Temperature mean**	**Precipitation mean**	**Temperature range**	**Precipitation range**	
1	√					√		141.41
2	√	√				√		143.10
3	√		√			√		144.07
4	√	√				√	√	145.29
5	√	√	√	√	√	√	√	150.72
6	√	√		√				151.13
7		√		√				152.79
8	√	√		√				158.47
9 (null model)								168.16

Table S2: Confusion matrix for the attribution of disturbance activity clusters from wood density and temperate range.

**Cluster (predicted)**	**Cluster observed**			**Commission error (%)**
	**Low**	**Moderate**	**High**	
Low	6	8	0	57.14
Moderate	12	17	6	51.43
High	0	9	19	32.14
Omission error (%)	66.67	50.00	24.00	

Table S3. Rules for conversion of LPJ-GUESS PFT distributions to biomes, adapted from Smith et al. (2014). Rules are applied sequentially in the order in the table.

**Rules (LAI = Leaf area index)**	**Biome name**
(LAI_{all_tree} > 2.0 & LAI_{bor_tree} > 0.8* LAI_{all_tree}) & (LAI_{BNE} > LAI_{BNS} | LAI_{IBS} > LAI_{BNE})	Needleleaf evergreen forest
(LAI_{all_tree} > 2.0 & LAI_{bor_tree} > 0.8* LAI_{all_tree}) & (LAI_{BNS} > LAI_{BNE} | LAI_{BNS} > LAI_{IBS})	Boreal deciduous forest
(LAI_{all_tree} > 2.0 & LAI_{temp_tree} > 0.8* LAI_{all_tree}) & LAI_{TeBE} > 0.5* LAI_{all_tree}	Temperate evergreen forest
(LAI_{all_tree} > 2.0 & LAI_{temp_tree} > 0.8* LAI_{all_tree}) & LAI_{TeBS} > 0.5* LAI_{all_tree}	Temperate deciduous forest
(LAI_{all_tree} > 2.0 & LAI_{temp_tree} > 0.8* LAI_{all_tree}) & LAI_{TeNE} > 0.5* LAI_{all_tree}	Needleleaf evergreen forest
LAI_{all_tree} > 2.0	Temperate/boreal mixed forest
LAI_{all_tree} > 0.5 & LAI_{all_tree} < 2.0 & LAI_{bor_tree} > 0.8* LAI_{all_tree} & (LAI_{BNE} > LAI_{BNS} | LAI_{IBS} > LAI_{BNS})	Needleleaf evergreen forest
LAI_{all_tree} > 0.5 & LAI_{all_tree} < 2.0 & LAI_{bor_tree} > 0.8* LAI_{all_tree} & (LAI_{BNS} > LAI_{BNE}| LAI_{BNS} > LAI_{IBS})	Boreal deciduous forest
LAI_{all_tree} > 0.5 & LAI_{all_tree} < 2.0 & LAI_{all_tree} > 0.8* LAI_{all}	Xeric woodland/shrubland
LAI_{all_tree} < 0.5 & LAI_{grass} > 0.2 & Latitude > 54.0	Tundra
LAI_{all} > 2.0	Grassland

Subscript meaning as follows: all = sum over all PFTs; all_tree = all tree PFTs; grass = all herbaceous PFTs; bor_tree = all boreal trees; BNE = boreal needleaved evergreen PFTs; BNS = boreal needleleaved deciduous PFTs; IBS = shade-intolerant broadleaved deciduous PFT; TeBE = temperate broadleaved evergreen PFT; TeNE = temperate needleleaved evergreen PFT. & and | are the AND and OR logical operators, respectively.

Table S4. Comparison of simulated stand-replacing disturbance return intervals with paleo and historical fire return intervals for suitable sites across the boreal zone.

**Study**	**Location[1]**	**Time period**	**Observed fire return interval (years)**	**Simulated disturbance return interval for 2001-2014 (years)**
Kelly et al. (2013)	65.75°N 146.25°W	Last 3000 years	111	179
Higuera et al. (2009)	67.25°N 151.25°W	Last 5500 years	135 (113-160)[2]	159
Higuera et al. (2009)	66.75°N 154.25°W	Last 5500 years	171 (135-216)[2]	159
Prince et al. (2018)	60.25°N 134.75°W	Last 12 000 years	120 (100-142)[2]	244
Senichi et al. (2015)	49.25°N 89.75°W	Last 5000 years	200	200
Kharuk et al. (2016)	65.25°N 99.75°E	Last 300 years	106 ± 36	186
Kharuk et al. (2008)	63.75°N 105.75°E	Last 300 years	82 ± 7	200
Kharuk et al. (2011)	66.25°N 99.75°E	Last 300 years	200 ± 51	192

[1] Locations are the closest LPJ-GUESS grid cell with forest.

[2] 95% confidence interval

Table S5. Assignment of species to LPJ-GUESS plant functional types.
(see supplementary spreadsheet)

References

Bergeron, Y., & Fenton, N. J. (2012). Boreal forests of eastern Canada revisited: Old growth, nonfire disturbances, forest succession, and biodiversity. *Botany*, *90*(6), 509–523. https://doi.org/10.1139/B2012-034

Haxeltine, A., & Prentice, I. C. (1996). BIOME3: An equilibrium terrestrial biosphere model based on ecophysiological constraints, resource availability, and competition among plant function types. *Global Biogeochemical Cycles*, *10*(5), 551–561.

Heinselman, M. L. (1981). Fire and Succession in the Conifer Forests of Northern North America. In D. C. West, H. H. Shugart, & D. B. Botkin (Eds.), *Forest Succession: Concepts and Application* (pp. 374–405). Springer.

Hengl, T., Walsh, M. G., Sanderman, J., Wheeler, I., Harrison, S. P., & Prentice, I. C. (2018). Global mapping of potential natural vegetation: An assessment of machine learning algorithms for estimating land potential. *PeerJ*, *2018*(8), 1–36. https://doi.org/10.7717/peerj.5457

Higuera, P. E., Brubaker, L. B., Anderson, P. M., Hu, F. S., & Brown, T. A. (2009). Vegetation mediated the impacts of postglacial climate change on fire regimes in the south-central Brooks Range, Alaska. *Ecological Monographs*, *79*(2), 201–219. https://doi.org/10.1890/07-2019.1

Kelly, R., Chipman, M. L., Higuera, P. E., Stefanova, I., Brubaker, L. B., & Hu, F. S. (2013). Recent burning of boreal forests exceeds fire regime limits of the past 10,000 years. *Proceedings of the National Academy of Sciences of the United States of America*, *110*(32), 13055–13060. https://doi.org/10.1073/pnas.1305069110

Kharuk, V. I., Dvinskaya, M. L., Petrov, I. A., Im, S. T., & Ranson, K. J. (2016). Larch forests of Middle Siberia: long-term trends in fire return intervals. *Regional Environmental Change*, *16*(8), 2389–2397. https://doi.org/10.1007/s10113-016-0964-9

Kharuk, V. I., Ranson, K. J., & Dvinskaya, M. L. (2008). Wildfires dynamic in the larch dominance zone. *Geophysical Research Letters*, *35*(1), L01402. https://doi.org/10.1029/2007GL032291

Kharuk, V. I., Ranson, K. J., Dvinskaya, M. L., & Im, S. T. (2011). Wildfires in northern Siberian larch dominated communities. *Environmental Research Letters*, *6*(4), 045208. https://doi.org/10.1088/1748-9326/6/4/045208

Lecomte, N., & Bergeron, Y. (2005). Successional pathways on different surficial deposits in the coniferous boreal forest of the Quebec Clay Belt. *Canadian Journal of Forest Research*, *35*(8), 1984–1995. https://doi.org/10.1139/x05-114

Marfo, J., & Dang, Q. L. (2009). Interactive effects of carbon dioxide concentration and light on the morphological and biomass characteristics of black spruce and white spruce seedlings. *Botany*, *87*(1), 67–77. https://doi.org/10.1139/B08-114

Niinemets, U., & Valladares, F. (2006). TOLERANCE TO SHADE, DROUGHT, AND WATERLOGGING OF TEMPERATE NORTHERN HEMISPHERE TREES AND SHRUBS. In *Ecological Monographs* (Vol. 76, Issue 4). http://mobot.mobot.org/W3T/Search/foc.htmli

Poulter, B., Aragão, L., Andela, N., Bellassen, V., Ciais, P., Kato, T., Lin, X., Nachin, B., Luyssaert, S., Pederson, N., Peylin, P., Piao, S., Pugh, T., Saatchi, S., Schepaschenko, D., Schelhaas, M., & Shivdenko, A. (2019). *The global forest age dataset and its uncertainties (GFADv1.1)*. https://doi.org/doi.pangaea.de/10.1594/PANGAEA.897392

Prince, T. J., Pisaric, M. F. J., & Turner, K. W. (2018). Postglacial reconstruction of fire history using Sedimentary charcoal and pollen from a small lake in southwest Yukon Territory, Canada. *Frontiers in Ecology and Evolution*, *6*, 209. https://doi.org/10.3389/fevo.2018.00209

Pugh, T. A. M., Lindeskog, M., Smith, B., Poulter, B., Arneth, A., Haverd, V., & Calle, L. (2019). Role of forest regrowth in global carbon sink dynamics. *Proceedings of the National Academy of Sciences of the United States of America*, *116*(10), 4382–4387. https://doi.org/10.1073/pnas.1810512116.

Senichi, D., Chen, H. Y. H., Bergeron, Y., & Ali, A. A. (2015). The effects of forest fuel connectivity on spatiotemporal dynamics of Holocene fire regimes in the central boreal forest of North America. *Journal of Quaternary Science*, *30*(4), 365–375.

Shorohova, E., Kuuluvainen, T., Kangur, A., & Jõgiste, K. (2009). Natural stand structures, disturbance regimes and successional dynamics in the Eurasian boreal forests: A review with special reference to Russian studies. In *Annals of Forest Science* (Vol. 66, Issue 2, pp. 201–201). EDP Sciences. https://doi.org/10.1051/forest/2008083

Smith, B., Wårlind, D., Arneth, a., Hickler, T., Leadley, P., Siltberg, J., & Zaehle, S. (2014). Implications of incorporating N cycling and N limitations on primary production in an individual-based dynamic vegetation model. *Biogeosciences*, *11*(7), 2027–2054. https://doi.org/10.5194/bg-11-2027-2014

Taylor, A. R., Gao, B., & Chen, H. Y. H. (2020). The effect of species diversity on tree growth varies during forest succession in the boreal forest of central Canada. *Forest Ecology and Management*, *455*, 117641. https://doi.org/10.1016/j.foreco.2019.117641

van Cleve, K., & Viereck, L. A. (1981). Forest Succession in Relation to Nutrient Cycling in the Boreal Forest of Alaska. In D. C. West, H. H. Shugart, & D. B. Botkin (Eds.), *Forest Succession: Concepts and Application* (pp. 185–211). Springer.
